# Role of cellular caspases, nuclear factor-kappa B and interferon regulatory factors in Bluetongue virus infection and cell fate

**DOI:** 10.1186/1743-422X-7-362

**Published:** 2010-12-06

**Authors:** Meredith E Stewart, Polly Roy

**Affiliations:** 1 Department of Pathogen Molecular Biology, London School of Hygiene and Tropical Medicine, Keppel Street, London WC1E 7HT, UK

## Abstract

**Background:**

Bluetongue virus (BTV) infection causes haemorrhagic disease in ruminants and induces cell death. The pathogenesis in animals and in cell culture has been linked to BTV-induced apoptosis.

**Results:**

In this report, we investigated BTV-induced apoptosis in cell culture in depth and show that both extrinsic (caspase-8 activation) and intrinsic (caspase-9 activation) pathways play roles in BTV apoptosis. Further, by using chemical inhibitors and knock-out cell lines, we show that these pathways act independently of each other in BTV infected cells. In addition to activation of caspase-8, -9 and executioner caspase-3, we also identified that BTV infection causes the activation of caspase-7, which results in the cleavage of poly (ADP-ribose) polymerase (PARP). BTV-induced cell death appears to be due to apoptosis rather than necrosis, as the HMBG-1 was not translocated from the nucleus. We also examined if NF-κB response is related to BTV-induced apoptosis as in reovirus. Our data suggests that NF-κB response is not linked to the induction of apoptosis. It is controlled by the degradation of only IκBα but not IκBβ, resulting in a rapid transient response during BTV infection. This was supported using an NF-κB dependent luciferase reporter gene assay, which demonstrated early response, that appeared to be suppressed by the late stage of BTV replication. Furthermore, virus titres were higher in the presence of NF-κB inhibitor (SN50), indicating that NF-κB has a role in initiating an antiviral environment. In addition, we show that BTV infection induces the translocation of interferon regulatory factors (IRF-3 and IRF-7) into the nucleus. The induction of IRF responses, when measured by IRF dependent luciferase reporter gene assay, revealed that the IRF responses, like NF-κB response, were also at early stage of infection and mirrored the timing of NF-κB induction.

**Conclusion:**

BTV triggers a wide range of caspase activities resulting in cell apoptosis. Although both NF-κB and IRF responses are induced by BTV infection, they are not sustained.

## Introduction

Bluetongue (BT) is a haemorrhagic disease of ruminants, which is caused by Bluetongue virus (BTV), a member of the orbivirus genus within the family *Reoviridae*. BTV consists of seven structural proteins (VP1 - VP7) organised in a double-capsid structure. Two of the seven proteins (VP2, VP5) make up the outer capsid of the virus and the remaining five proteins (VP1, VP3, VP4, VP6 and VP7) are located in the inner capsid or core together with the double-stranded RNA genome consisting of ten segments. Three non-structural proteins that are not associated with the virion are also expressed (NS1-3) in the infected cells. To date, 24 different serotypes have been officially recognised and an additional serotype has recently been identified by sequence analysis [1,2]. BTV is an insect-borne virus, which is transmitted from animal to animal by blood feeding midges (*Culicoides *spps) and has been endemic mainly in tropical and sub-tropical countries. Although BTV infects a wide variety of domestic and wild ruminants, classically, BT is considered predominantly as a sheep disease and indeed BTV infection in certain breeds of sheep may cause severe morbidity and high mortality. In recent years, BTV has emerged in northern Europe and re-emerged in the Mediterranean basin causing severe disease and high mortality in naïve ruminant populations. Outbreaks have affected not only sheep, but also other livestock such as cattle and goats [3,4]. The clinical symptoms of BTV infection are thought to be associated with virus-induced vascular injury and endothelial cell-derived inflammatory responses [5-8] and apoptosis [9], although host responses at a cellular level that result in the pathogenesis caused by BTV infection have not been investigated thoroughly.

BTV induces apoptosis both in cultured cells and in target tissues *in vivo *and one current hypothesis is that apoptosis plays a major role in the pathogenesis of BTV infection [10-12]. Virus infected cells that undergo apoptosis show highly characteristic morphological changes, including shrinkage, blebbing of the plasma membrane, chromatin condensation and DNA fragmentation. In a previous report, we showed that extracellular treatment with a combination of both the cellular receptor binding protein VP2 and the cell penetration protein VP5, is sufficient to trigger apoptosis through the activation of executioner caspase-3 [11]. Subsequent to this report, others have reported that both the extrinsic and intrinsic pathways are involved in the induction of apoptosis by BTV [9,10,12]. However, the results in these reports have contradictory conclusions, particularly in relation to caspase-8 activation. While Li *et al. *[10] reported that BTV infection does not cause caspase-8 cleavage [10], a subsequent publication by others presented the cleavage data of caspase-8 [12]. Further, the interrelationship between the intrinsic and extrinsic pathways in triggering apoptosis has not been investigated.

Previously, we have also identified the translocation of NF-κB into the nucleus from cytoplasm during BTV infection of mammalian cells and we had postulated, based on the finding with reovirus [13] that NF-κB activation by BTV infection was involved in induction of cellular apoptosis [11]. Activation of NF-κB by a viral infection promotes the expression of a variety of genes that are involved either in regulating the host survival immune responses or in apoptosis. However, certain virus infection such as African swine fever virus inhibits NF-κB activation, which results in enhancement of virus replication and thereby contributing to virus-induced pathogenesis [14].

Activation of NF-κB is generally a rapid response to an inducer, including virus infection [15]. The NF-κB exists as a heterodimer which is sequestered in the cytosol of unstimulated cells via non-covalent interactions with a class of inhibitor proteins, called IκBs (*viz*., IκBα, β, γ etc; [16]). These inhibitor proteins mask the nuclear localisation signal of NF-κB. Signals that induce NF-κB activity cause the phosphorylation of IκBs, their dissociation and subsequent degradation, allowing NF-κB proteins to enter the nucleus and induce gene expression. The specific phophorylation and degradation of the IκBs determines whether the activation of NF-κB is a rapid and transient response. In this report, we investigated the activation and role of NF-κB during BTV infection, demonstrating that it has a role in initiating an antiviral environment as a part of the innate immune response.

Another mechanism of the innate immune system in response to virus infection is the activation of interferon regulatory factors (IRF) that results in the secretion of antiviral cytokines, particularly, interferon (IFN). Since BTV induces strong cytokine responses, BTV may also trigger innate immune pathways via IRF that are responsible for regulating cytokine production [17-19]. In particular, IRF-3 is ubiquitously expressed and accumulates in the cytosol to enable a rapid response to viral infection and up-regulate the type 1 IRF [18]. Although the induction of IRF in the other members of the family *Reoviridae *has been documented, to date there is no published report on IRF activities in BTV infection.

In this study, we have examined the interrelationship between the intrinsic and extrinsic pathways of apoptosis in BTV infected cells and demonstrated that the intrinsic and extrinsic apoptotic pathways are independently triggered, and a second executioner caspase, caspase-7, is activated. The role of apoptosis or necrosis in the development of BTV pathogenesis is currently not determined, however, it has been suggested by some that it is due to cell necrosis [6] while others believe that cell apoptosis is the major cause for the disease [9]. The data that we obtained here demonstrates that BTV infection triggers strong apoptotic response indicating that it has some role in the disease. Since translocation of high mobility box group-1 (HMBG-1) from nucleus to cytoplasm is a distinct feature of necrosis, we examined if HMBG-1 was translocated during BTV infection [20-22]. The data demonstrated that HMBG-1 was not translocated from the nucleus thereby indicating that in cell culture apoptosis is the predominant cause of cell death. We also present evidence that NF-κB, although is activated, is unlikely to be responsible for BTV apoptosis. Indeed both BTV replication and cytopathic effect are enhanced in the presence of a chemical inhibitor of NF-κB activation. These results suggest that NF-κB activation may be involved in the development of the cytokine response. In addition, we observed that IRFs, particularly IRF-3, were up-regulated during BTV infection. Thus, it is likely that BTV infection triggers the NF-κB and IRF activations to establish an antiviral state in order to control BTV replication.

## Results

### Activation of cellular caspases in BTV infected cells

**Figure 1:**
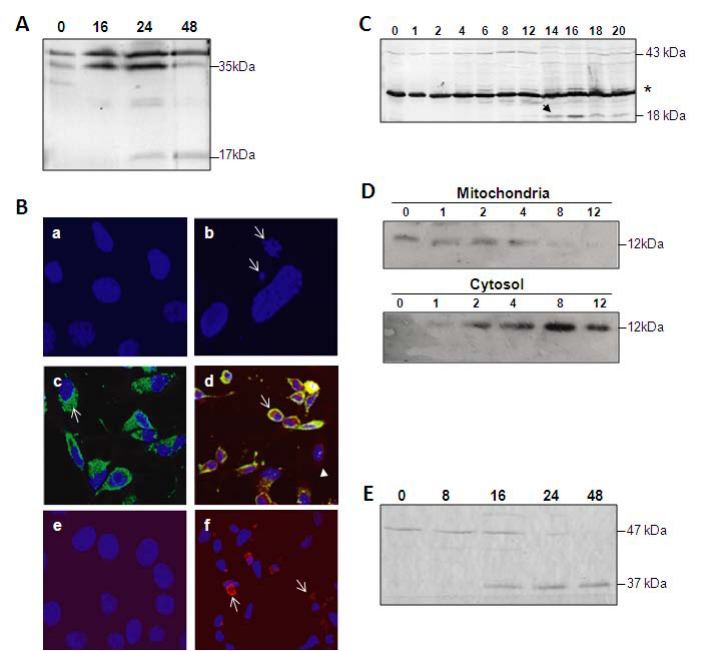
**Activation of apoptosis by intrinsic and extrinsic pathways in BTV infected mammalian cells**. **A: **Detection of cleavage of caspase-3 in BTV-1 infected HeLa cells at different times p.i. **B: **Immunofluorescence of BTV-1 infected HeLa cells shows the morphological changes associated with apoptosis. (a) Uninfected cells stained with Hoescht, (b) BTV-1 infected stained with Hoescht, the nuclear blebbing and condensation is indicated by a white arrow, (c) BTV-1 infected stained with Hoescht (blue) and immunofluorescence detection of VP5 (green) and (d) BTV-1 infected stained with Hoescht (blue), VP5 (green) and caspase-3 (red) detected by immunofluorescence. BTV infected HeLa cells undergoing apoptosis are indicated by the open arrow and uninfected cell indicated by the closed arrow. (e) Uninfected HeLa cells and (f) BTV-1 infected HeLa cells stained with Hoescht (blue) and anti-active caspase-8 monoclonal clonal antibody (red). Cleaved caspase-8 is visible in the BTV infected cells (open arrow) **C: **Detection of caspase-8 cleavage in BTV-1 infected HeLa cells at different time p.i. The procaspase-8 (43 kD), cleaved caspase-8 (18 kDa) product and a 26 kDa non-specific band (*) are indicated. **D: **Detection of cytochrome C (12 kDa) in the mitochondrial (top) and cytosolic (bottom) fractions of BTV-1 infected cells at different times post infection, demonstrating the release from the mitochondria and its accumulation in the cytosol. **E**: Western immunoblot analysis of caspase-9 cleavage in the whole cell lysate of BTV-1 infected cells. The procaspase-9 (47 kDa) and cleaved (37 kDa) products of caspase-9 are indicated.

Previous reports from our laboratory and by others have demonstrated that caspase -3, -8 and -9 are activated during infection with BTV [9-12]. To further characterise the activation of apoptotic pathways during BTV infection, HeLa cells were infected with BTV-1 and activation of caspase-3, -8 and -9 were monitored by western immunoblot analyses. Activation of all three caspases, as expected, was observed during BTV infection in both cell types (Figure 1). Caspase-3 is one of the executioner molecules of the caspase cascade that leads to apoptosis. The cleavage of the full-length 35 κDa procaspase-3 to produce the active 17 κDa caspase-3 product was detected after 24 h post infection (p.i.) in BTV infected mammalian cells, and was continually detected over the time course of infection (Figure 1A). To substantiate the biochemical data, BTV-1 infected HeLa cells were fixed at 24 h p.i. and stained with Hoescht in order to visualise the morphological changes in infected cells (Figure 1B). Distinct chromatin condensation and nuclear fragmentation were observed in BTV infected cells (Figure 1B, a) indicating signs of apoptosis. There was no condensation or fragmentation in the nucleus of the uninfected control cells (Figure 1B, b). To confirm that morphological changes were specific to BTV infected cells we performed immunofluorescence, anti-VP5 antibody was added onto the infected cells after permeabilisation and cells were stained with Hoescht. The results showed early stages of nuclear condensation and fragmentation in BTV infected cells (Figure 1B, c), and not in the bystander uninfected cells in the same sample. Further, dual labelling of infected cells with anti-VP5 antibody and anti-caspase-3 antibody confirmed that apoptosis occurred in infected cells, whilst none of the bystander cells showed any signs of apoptosis (Figure 1B, d).

Previously, we have demonstrated that treatment of mammalian cells with only the two outer capsid proteins (VP2 and VP5) resulted in the induction of apoptosis, which led us to hypothesise that the virus attachment proteins trigger apoptosis via the extrinsic pathway. To investigate the role of the extrinsic pathway in triggering BTV apoptosis, we examined the cleavage of caspase-8. Whole cell lysate from BTV infected cells was monitored for the cleavage of caspase-8 by western immunoblot. The 18 kDa cleavage product was observed from 12-14 h onwards and reached a maximum at 16 h p.i. (Figure 1C). To further investigate that caspase-8 was active, we performed immunofluroscence microscopy assay using a specific antibody that recognises the active caspase-8 protein. HeLa cells infected with BTV at a low MOI were fixed at 24 h p.i. with paraformaldehyde, permeabilised and treated with anti-caspase-8 followed by anti-rabbit Alexa Fluor 488. The active form of caspase-8 was observed only in the cells infected with BTV and not in the control uninfected cells (Figure 1B, e &1B, f).

In the intrinsic pathway, apoptosis is triggered by internal signals leading to leakage of the outer mitochondrial membrane, which causes the release of cytochrome C into the cytoplasm. We examined if BTV infection causes mitochondrial damage and the release of cytochrome C. Both cytosol and mitochondria fractions of BTV-infected HeLa cells were examined at different times by western analysis, using anti-cytochrome C polyclonal antibody. As shown in Figure 1D, the cytochrome C was detected in the cytosol as early as 2 h p.i. and by 8 h p.i. it was translocated entirely to the cytosol from the mitochondria. To determine further the activation of the intrinsic pathway by BTV infection, the cleavage of procaspase-9 to the active form was examined. The 47 kDa procaspase-9, which mediates the activation of apoptosis via the mitochondrial pathway, was cleaved to the activated 35 kDa fragment after 8 h p.i. (see Figure 1E). The release and timing of cytochrome C depletion from the mitochondria coincided with caspase-9 activation. This would indicate that the release of this protein from the mitochondria into the cytoplasm was due to the BTV-induced apoptosis.

As the cleavage of caspase-8 and caspase-9 occurred at similar times p.i., we examined whether the mitochondrial damage and subsequent release of cytochrome C into the cytosol could also be mediated by the cleavage of BID (a death agonist member of the Bcl2/Bclx_L _family; [23,24]) via caspase-8 activation. This would indicate that there is a cross-talk between the intrinsic and extrinsic pathways. The truncated BID (tBID) product was not detected in any of the BTV infected cells by western analysis (data not included). Thus, caspase-9 activation is likely to be independent of caspase-8. The accumulated data suggests that both the intrinsic and extrinsic pathways are activated and the intrinsic pathway is independent of the extrinsic pathway in BTV infected mammalian cells.

### Caspase activations by intrinsic and extrinsic pathways are independent in BTV infection

**Figure 2:**
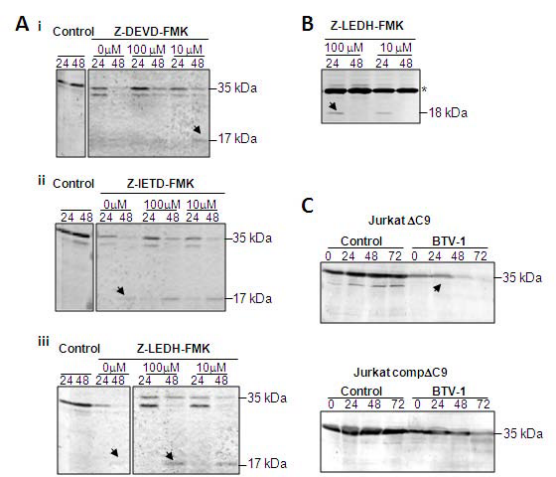
**Cleavage of caspase-3 by BTV-1 infection either in inhibitor-treated cells or in knock-out cells**. **A: **Immunoblot of Caspase-3 using anti-caspase-3 polyclonal antibody (Abcam: ab17819). **B: **The cleavage of caspase-8 in caspase-9 inhibitor (z-LEHD-FMK) treated cells infected with BTV-1. The 18 kDa cleaved product and a 26 kDa non-specific band (*) are indicated. **C: **Immunoblot of capase-3 cleavage products at different times of BTV-1 infection in caspase-9 deficient Jurkat T-cells (JurkatΔC9) and complemented caspase-9 deficient Jurkat T-cells (Jurkat compΔC9) using an alternate anti-caspase-3 polyclonal antibody (Abcam: ab90437). Representative results from three independent experiments are shown.

The interrelationship between the intrinsic and extrinsic pathways and induction of BTV dependent apoptosis was further investigated using specific chemical inhibitors for each caspase-3, -8 and -9 activities at different concentrations. The ability of the chemical inhibitors to interrupt the activation of caspase-3 after BTV infection was monitored. HeLa cells were treated with various chemical inhibitors of caspases 1 h prior to BTV infection, harvested at 24 and 48 h p.i. and whole cells lysates were analysed for the cleavage of caspase-3 (Figure 2A). Since caspase-3 cleavage was observed from 24 h p.i. onwards (see Figure 1), earlier time points were not monitored. Both control uninfected and BTV infected cells were also grown in the presence of DMSO as used for the inhibitors. The cleavage of procaspase-3 (35 κDA) was delayed in the presence of caspase-3 specific inhibitor (Z-DEVD-FMK, Figure 2A, i) as expected, and at the highest concentrations of the inhibitor, the cleavage was not observed at 24 h p.i. In contrast, in the absence of the inhibitor, procaspase was cleaved and the 17 κDA cleavage form at 24 h p.i. was clearly identified by western blot (Figure 2A, i). Caspase-3 cleavage was also detected in BTV infected HeLa cell samples that had been treated with either Z-IETD-FMK (specific capase-8 inhibitor) or Z-LEHD-FMK, (specific caspase-9 inhibitor) at 24 and 48 h p.i (Figure 2A, ii &2A, iii).

There was no apparent difference in the cleavage of caspase-3 in BTV infected cells in the presence or absence of either the caspase-8 or caspase-9 specific inhibitors. These inhibitor studies further support the independent activation of both intrinsic and extrinsic pathways of apoptosis by BTV infection.

Since there are contradictory reports regarding the activation of caspase-8 in bluetongue infections [10,12,25], we determined if caspase-8 activity was present independently of caspase-9 activity during BTV infection. Western analysis of BTV infected cells showed the cleaved products of the activated caspase-8 in infected cells treated with the caspase-9 inhibitor (Z-LEHD-FMK; Figure 2B). At 24 h p.i. cleavage of caspase-8 was observed in the presence of Z-LEHD-FMK inhibitor at all concentrations tested. There was no cleavage in the 48 h p.i. samples, which is consistent with the control BTV infected cells. The data added further support to the notion that the pathways are independent in BTV infection.

To substantiate the data obtained with chemical inhibitors, we infected a caspase-9 deficient cell line (Jurkat ΔC9) with BTV and monitored caspase 3 cleavage. The 35 kDa procaspase-3 is cleaved in the BTV infected Jurkat ΔC9 from 48 h p.i. (Figure 2C). This antibody is specific for the procaspase but not the cleaved product. There was a slight delay in procaspase-3 cleavage in the complemented-Jurkat ΔC9 with cleavage observed at 72 h p.i. The results were consistent with the pharmacological data demonstrating that caspase-3 could still be activated in absence of caspase-9 activation (Figure 2C). Thus, both pathways are activated during BTV infection but are not dependent on each other.

### Caspase-7 is activated during BTV infection

**Figure 3:**
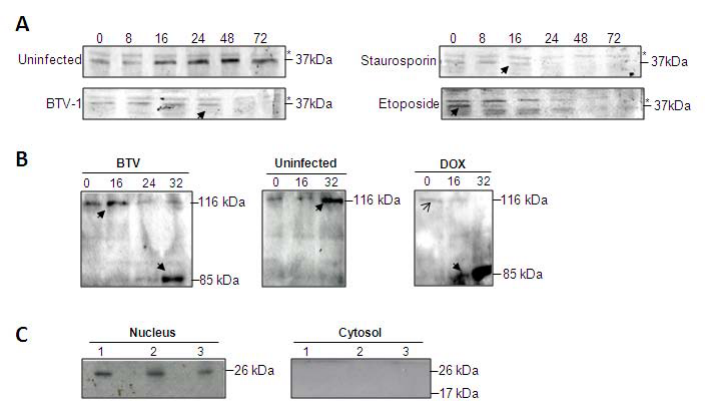
**BTV causes an apoptotic response in preference to a necrotic response in mammalian cell culture**. **A: **The detection of a second executioner, caspase-7, in BTV-1 infected cells. Whole cell lysate extracts were prepared from different times post infection and monitored for caspase-7 cleavage with a specific anti-caspase-7 polyclonal antibody. **B: **Detection of caspase-dependent PARP cleavage in BTV infection by immunoblot at different times p.i. Cleavage of the PARP from the full length (116 KDa) to inactive cleaved form (85 kDa) is observed in the BTV infected and DOX treated cells and not the control uninfected. **C: **Detection of HMGB-1 (26 kDa) by immunoblot in the nuclear (left) and cytosolic (right) fractions of HeLa cells that were uninfected (1), DOX-treated (2) and BTV-1 infected (3).

Caspase-8 and -9 are not only responsible for the cleavage of caspase-3, but also cleave caspase-7. We examined if caspase-7 was also cleaved in BTV infected cells. Jurkat T-cells were infected with BTV-1, and cell lysate harvested at various times were subjected to western analysis. The 35 kDa procaspase-7 was clearly visible in the control, uninfected cells, but was not detectable in BTV infected cells at 16 h p.i. (Figure 3A). Distinct cleavage and reduction of the procaspase-7 form were also evident in the positive control cells that were treated either with chemical inducers staurosporin or with etopside at 8 h and 16 h post-treatment, respectively (Figure 3A). The use of a second executioner caspase indicates that BTV induces a robust apoptotic response in infected cells.

### BTV-1 infection induces the caspase-dependent cleavage of PARP

The executioner caspases-3 and -7 initiate the later stages of apoptosis by inactivating key host cell proteins including PARP (poly (ADP-ribose) polymerase), which is involved in DNA repair.

Inactivation of PARP by cleavage results in DNA fragmentation. We investigated if PARP is also cleaved during BTV infection. Mammalian cells were infected with BTV-1 and PARP cleavage was monitored at different times post-infection by western analysis using specific antibody (Figure 3B). The full-length 116 KDa PARP could be detected from all samples at 0 to 16 h p.i. in BTV infected cells, and the 85 kDa cleavage product was detectable from 24 h p.i. onwards (Figure 3B). In contrast to BTV infection, a chemical inducer of PARP (DOX, Doxorubicin) induced the cleavage of PARP in the cell culture as early as 16 h after its addition (Figure 3B).

As cleavage of PARP occurs only in apoptotic cells and to further support the notion that BTV infection of mammalian cells primarily results in apoptosis rather than necrosis, the translocation of HMBG-1 protein from the nucleus to the cytoplasm was investigated. HMBG-1 translocation is considered to be an indicator of a necrotic state rather than an apoptotic state of the cells [26]. BTV infected cells were harvested at 24 h p.i. and the cytosolic and nuclear fractions were separated and analysed by western analysis. It was clear that HMBG-1 was retained in the nuclear fraction and there was no detectable translocation of the protein to the cytosolic fraction during BTV infection (Figure 3C).

### NF-κB has a limited role in BTV induced apoptosis

**Figure 4:**
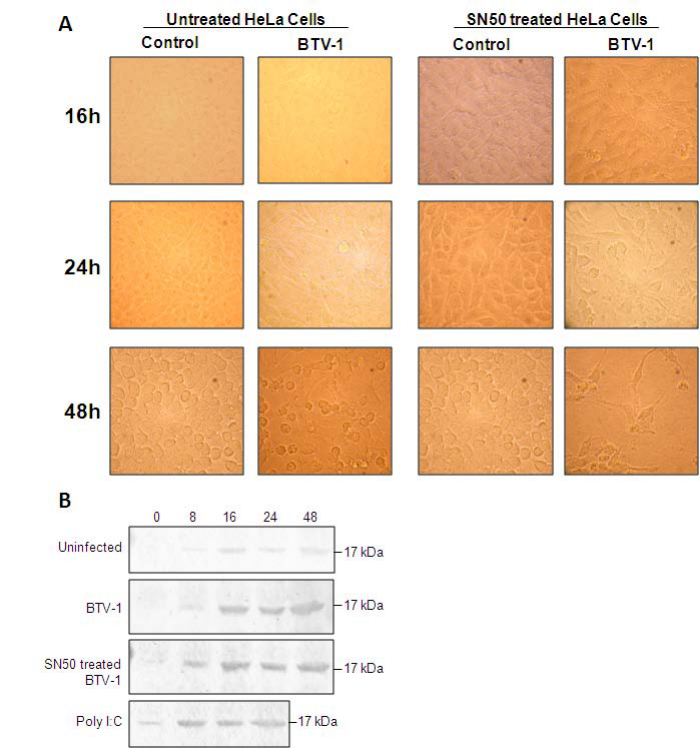
**Inhibition of NF-κB with SN50 inhibitor does not prevent apoptosis in BTV infected cells**. **A: **Morphological changes associated with BTV infection in the presence or absence of SN50 inhibitor at different times p.i. BTV infected cells and uninfected cells without the inhibitor and BTV infected cells and uninfected cells in the presence of the SN50 inhibitor are also shown. **B: **NF-κB induction by BTV-1 infection of mammalian cells does not influence the cleavage of caspase-3. Immunoblot analysis of BTV-1 infected, BTV-1 infected that had been pre-treated with 32 uM of SN-50 inhibitor, uninfected and poly I:C treated HeLa cells was undertaken on whole cell lysate at different times p.i and the cleavage of caspase-3 detected with rabbit anti-caspase-3. The cleaved 17 kDa product is shown.

NF-κB is generally involved in cellular responses not only to stress, injury and cytokines but also to virus infection. In particular, it plays a key role in regulating the innate immune responses to virus infection. Conversely, it may also be involved in triggering inflammation and cell death (i.e., apoptosis). Thus, it controls the balance between cell survival and death. We have shown previously that NF-κB is translocated to the nucleus during BTV infection [11] indicating that BTV infection causes the activation of the NF-κB complex and hypothesised that it might be involved in inducing apoptosis similar to reoviruses [13]. Therefore, we investigated whether activation of NF-κB in BTV infection is indeed involved in the induction of apoptosis. HeLa cells were treated with 32 μM NF-κB inhibitor (SN50) for 60 min prior to infection with BTV and cell morphology was monitored by light microscopy. Typical BTV induced cytopathic effect was observed in cells treated with SN50 at earlier time points (24 h p.i., Figure 4A) in comparison to the normal BTV infection (48 h p.i., Figure 4A). The visible cytopathic effect observed in the treated and untreated BTV infected cells would indicate that NF-κB is not involved in the morphological changed induced by the virus. To further examine if NF-κB is involved in the induction of apoptosis, we tested the cleavage of caspase-3 in the presence of SN50 during BTV infection. There was no significant difference in the caspase-3 cleavage activity of BTV-1 infection between the SN50 treated versus the untreated cells (Figure 4B). These results indicate that NF-κB response to BTV-1 infection is probably similar to type 1 reovirus strain Lang (T1L, [27]), where NF-κB activation has a minimal role in the induction of apoptosis. Our data would indicate that NF-κB activation in BTV infected cells is not directly involved in the induction of apoptosis.

### NF-κB response is regulated by IkBα degradation during BTV infection

**Figure 5:**
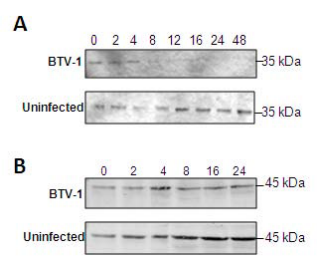
**BTV-1 infection of HeLa cells leads to the degradation of IkBα and not IkBβ**. Whole cells fractions from HeLa cells infected with BTV-1 (MOI-1) at different times p.i. were analysed by immunoblot using specific antibodies **A: **IkBα immunoblot of BTV-1 infected cells and uninfected cells at different times p.i. **B: **Immunoblot of IkBβ in BTV-1 infected and control uninfected cells.

The response of NF-κB during BTV infection was further characterised. The type of response and activation of NF-κB to an infection is controlled by the IκB complex, which mask the nuclear translocation signals and act as inhibitor proteins. We investigated the phosphorylation and degradation of IκBα and β in HeLa cells infected with BTV in order to analyse the NF-κB response. Cell lysates were harvested and subjected to western blot using specific antibodies for IκBα and IκBβ. While IκBα degradation was clearly detected in BTV infected cells (Figure 5A), there was no detectable degradation of IκBβ (Figure 5B). The phosphorylation and degradation of the 35 kDa IκBα was rapid in the BTV infected cells and only up to 4 h p.i. IκBα could be observed (Figure 5A), while the chemically (DOX) treated cells when used as positive controls, showed a similar pattern of degradation to that of BTV infection (data not shown). There was no degradation of IκBα in the uninfected cells (Figure 5A). In contrast, IκBβ subunit degradation was not observed in the BTV-1 infected samples at the time points monitored (Figure 5B). Such selective degradation of IκBα, but not IκBβ by BTV infection means that it triggers the typical rapid response of NF-κB in mammalian cells rather than a persistent activation. The results indicate that the nature of the NF-κB response may be rapid.

### Activation of NF-κB response is early during BTV infection

**Figure 6:**
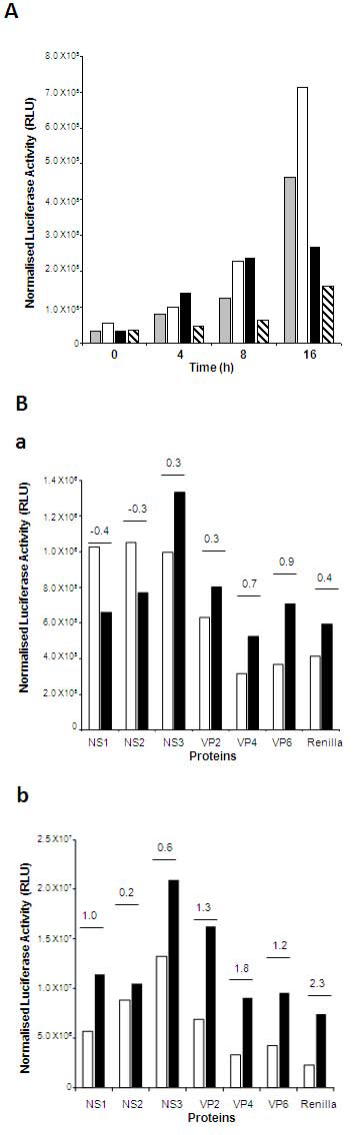
**BTV-1 infection of HeLa cells activates NF-κB response**. **A: **HeLa cells were transfected either pGL3Basic luciferase control or pGL3NFκB luciferase and infected with BTV-1 (MOI 0.5-1) or treated with DOX and analysed for luciferase activity (RLU). Normalised RLU values for the control uninfected (grey), DOX treated (white), BTV-1 (black) and BTV-1 in the presence of SN50 inhibitor (black-lined) at different time points (0 h-16 h) are shown. **B: **Expression of luciferase (RLU) from an NF-κB dependent luciferase reporter in the presence of individual BTV proteins after induction with poly I:C assay at two different times, 6 h (a) and 24 h (b). The normalised RLU was measured in uninduced (white) and induced (black) and the fold induction (<0 = suppression, 0 = no change, >0 = induction) was calculated as indicated. This is a representative experiment and the data presented is normalised from 3 different tests.

The degradation of IκBα and translocation of the NF-κB into the nucleus during BTV infection prompted us to investigate the level of NF-κB activation using a NF-κB dependent luciferase reporter gene system. Up-regulation of the NF-κB dependent transcripts was detected in BTV infected cells from 4-8 h p.i. (Figure 6A). When relative light units of firefly luciferase (RLU) activities were normalised from three different experiments, there was clear indication of ~2-3 fold NF-κB activation in BTV infected cells in comparison to that of the controls, (uninfected cells, Figure 6A). However, after the peak activation at 8 h p.i., the activity was effectively suppressed. The NF-κB response for all experiments showed a similar pattern. Furthermore, when SN50 inhibitor treated cells were infected with BTV, the RLU at each time point was lower in comparison to the uninfected cells (Figure 6A). The data indicated that although NF-κB is active during BTV infection, the response is early and transient.

### BTV-1 proteins can suppress the activation of NF-κB signal pathway

Since the NF-κB dependent reporter gene assay indicated the suppression of the NF-κB activation by BTV replication, we undertook a preliminary study to examine the involvement of each viral protein in NF-κB activation. In order to measure the effect of the newly synthesised individual proteins on the induction or suppression of the NF-κB response, HeLa cells were co-transfected with the NF-κB dependent reporter plasmid together with various BTV mRNAs, S2 (VP2), S4 (VP4), S5 (NS1), S8 (NS2), S9 (VP6) and S10 (NS3) that were synthesized from exact copy T7 vectors as described in Materials and Methods. The transcripts were co-transfected with the reporter plasmid to reduce the effect of mRNA triggering NF-κB response. NF-κB activation was induced with poly I:C and the firefly luciferase activity was measured at 6 and 24 h time intervals post-induction. To ensure that the mRNA did not induce a response and that the suppression of poly I:C inducted NF-κB response was BTV specific, we used a T7 generated *Renilla *luciferase mRNA as a control since the protein can be distinguished from the firefly luciferase. At 6 h post-induction, only NS1 and NS2, but not NS3 or the structural proteins had an inhibitory effect on the poly I:C induction of NF-κB response (Figure 6B). At 24 h post-induction of NF-κB by poly I:C, the suppressive effect of the NS1 diminished with an increase in the RLU, in comparison to the NS2 sample, which only had a slight increase in the RLU measure (Figure 6B). This indicates that both NS proteins have a role in controlling the NF-κB response to BTV infection.

### NF-κB activation controls early virus replication in mammalian cells

**Figure 7:**
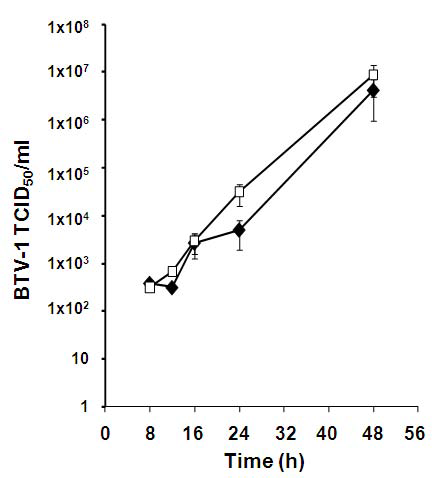
**Replication of BTV-1 in HeLa cells in the presence of a NF-κB inhibitor**. BTV growth in the presences of 32 μM NF-κB inhibitor (□) and mock treated (◆) HeLa cells at different times p.i was determined by TCID_50_/ml using Muench and Reed method. Representative data from two independent assays undertaken in triplicate is presented.

The accumulated data so far indicates that NF-κB activation in response to BTV infection is an early and transient response and appears to have a minimal role in the induction of apoptosis. Therefore, we wanted to examine if it was involved in limiting virus replication. Cells were treated with 32 μM SN50 inhibitor prior to infection with BTV-1 (~0.1-0.5 MOI) and the virus titres at various times were determined by tissue culture infectious dose of 50% (TCID_50_/ml). During the early phase of virus growth (8, 12 and 16 h p.i.), there was a minimal difference in the virus titres between the SN50 treated and untreated cells (Figure 7). However, at 24 h p.i., a significant difference was detected in the virus titres between the untreated versus the inhibitor treated cells. In the presence of the inhibitor, virus titres were significantly higher (p > 0.1, 2 replicates repeated duplicate by TCID_50_/ml). This effect is diminished at the 48 h and yielded very similar titres in both treated and untreated cells (Figure 7). This would be consistent with the observation that BTV and some of the virus proteins specifically suppress the NF-κB at later times p.i. to enable efficient virus progeny to be produced. The BTV-1 titres were consistently higher when the virus was grown in the presence of the SN50 inhibitor, which further supports our hypothesis that during BTV infection of mammalian cells, NF-κB activation aims to suppress replication of the virus. This observation together with the other data that we obtained indicates that the main role of NF-κB during BTV infection is to suppress virus replication.

### Interferon regulatory factors are up-regulated during BTV-1 infection

**Figure 8:**
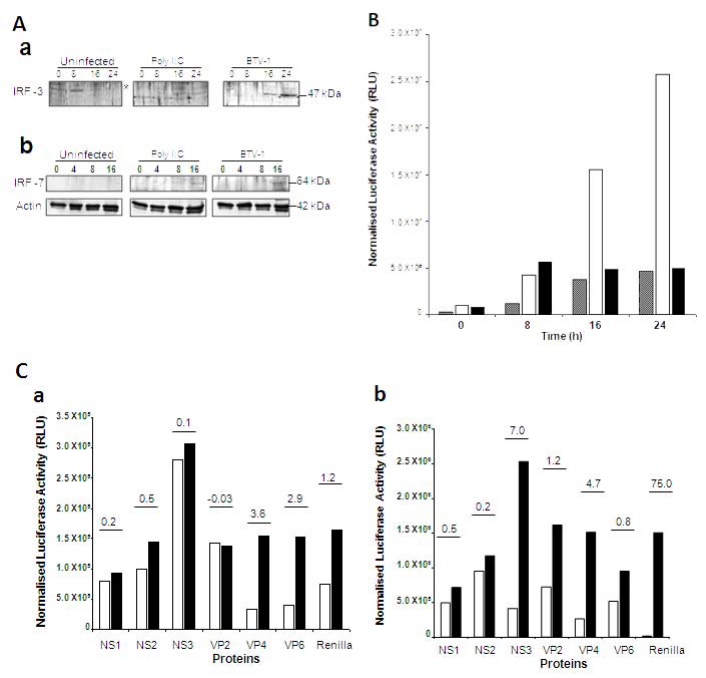
**BTV-1 infection of HeLa cells activates an IRF response**. **A:**Nuclear translocation of IRF-3 (a) and IRF-7 up-regulation (b) in BTV-1 infected HeLa cells were detected by immunoblots at different times p.i. **B: **HeLa cells were transfected with IRF dependent firefly luciferase reporter and infected with BTV-1 (MOI 0.5) or transfected with poly I:C and analysed for luciferase activity (RLU). Normalised data for the control uninfected (grey), poly I:C treated (white), and BTV-1 infected (black) at 4 different times are shown. **C: **Expression of luciferase (RLU) from an IRF dependent firefly reporter in the presence of individual BTV proteins after induction with poly I:C at two different times, 6 h (a) and 24 h (b). The normalised RLU for each protein in induced (black) and uninduced (white) samples and the fold induction (< 0 = suppression, 0 = no change, >0 = induction) are shown. This is a representative experiment and the data presented is normalised from 3 different tests.

The results so far indicate that the role of NF-κB during BTV infection probably relates to induction of an antiviral state of infected cells. Indeed, it has already been documented that BTV causes a strong cytokine response during early infection both *in vivo *and *in vitro *[5-7,9,28,29]. Infection by viruses or transfection with dsRNA results in rapid induction of type 1 IFN that establishes the innate immune response and requires multiple transcriptional proteins [30]. Since the interferon regulatory factor-3 (IRF-3) contributes to a first line of defence against virus infection, we investigated whether the BTV infection of mammalian cells results in the activation of IRF-3 and related IRF-7. HeLa cells were infected with BTV, and samples from whole cell lysate and cell fractions were harvested at different times p.i. and subjected to western analysis. Translocation of IRF-3 into the nucleus of BTV infected cells was detected after 8 h p.i. (Figure 8A, a). The up-regulation and translation of IRF-7 in response to BTV-1 infection of HeLa cells was observed in the whole cell lysate at 16 h p.i. (Figure 8A, a).

The activities of IRF-3 and -7 by BTV were further verified using an IRF dependent firefly luciferase reporter gene system to measure the activation as described for the activation of NF-κB above. The data was normalised from at least 3 independent replicates. The firefly luciferase RLU values in BTV infected cells at 8 h p.i were higher than both controls, the poly I:C transfected and the uninfected cells (Figure 8B). Activation of the IRF-dependent reporter by BTV infection had a peak activity at 8 h and greater than 2-fold increase from the uninfected control cells (Figure 8B). The IRF response to BTV infection followed a similar trend for all experiments undertaken. The poly I:C strongly activated the IRF dependent reporter as expected (Figure 8B).

### Virus proteins play a role in limiting the induction of IRF response

The response observed from the IRF dependent luciferase activity during BTV infection demonstrated similar kinetics to the NF-κB dependent firefly luciferase activity and therefore it is likely that *de novo *synthesis of intracellular BTV proteins play a role in limiting the IRF response. An experiment analogous to the NF-κB, using T7 mRNA for various BTV proteins together with an IRF dependent firefly luciferase report construct was undertaken to determine if the IRF response was indeed controlled by newly synthesised BTV proteins when induced with poly I:C. Interestingly, at 6 h post-transfection, unlike the NF-κB response, not only did all the three NS proteins, but also the major outer capsid protein VP2 had an effect on the poly I:C induced IRF activity (Figure 8C) in comparison to the expressed Renilla lucerifase control. The two internal catalytic proteins, VP4 and VP6, failed to reduce the poly I:C induced IRF response. At 24 h post-induction, only NS1 and NS2 had some inhibitory effect, while an increase in RLU was observed for VP2 (~1.3 fold) and NS3 (~7 fold). These results suggest that with the increase of BTV proteins synthesis, activation of IRF decreases in order to allow BTV replication.

## Discussion

The infection of mammalian cells by many viruses induces apoptosis and a variety of signal transduction pathways either to promote cell survival or to enhance the cell death. BTV infection of mammalian cells also triggers apoptosis. There are two common pathways for the induction of apoptosis and it appears that BTV may trigger both intrinsic and extrinsic pathways [9-12]. The extrinsic pathway is primarily initiated by virus attachment to receptors, while the intrinsic pathway is mediated by damage to the mitochondria. In this report, we have undertaken a series of stepwise experiments to examine the cellular activations of various caspases and thereby induction of apoptosis during BTV infection. Further, we investigated if the caspase activities via intrinsic and extrinsic pathways during BTV infection are interdependent. Data obtained in this report conclusively confirmed the activation of caspase-8 in BTV infected cells by two different methods. The cleavage of caspase-8 was observed from 12 h p.i. by western analysis and the active, cleaved form was visualised by confocal microscopy. As the primary activation of caspase-8 is due to receptor binding, it was expected that caspase-8 activation would occur relatively early after infection with BTV. However, we noted that the caspase-8 activation in BTV infection was somewhat delayed compared to other viruses (e.g., reoviruses). Although we have not investigated further, it can be hypothesised that it is regulated by c-FLIP, an apoptosis inhibitor, as in reovirus infection [27]. The control of caspase-8 by c-FLIP could be regulated by the activity of MAPK p38 [31,32], which has been shown to play a role in BTV-induced apoptosis and endothelial damage [33,34].

Our data also demonstrated the involvement of the intrinsic pathway in BTV infection as documented by the disruption of the mitochondria and release of cytochrome C which is known to be responsible for activating a number of events that leads to the cleavage of caspase-9. Thus, our data supports the recent report that cytochrome C is released from the mitochondria during an early stage of the BTV replication cycle [10]. The direct evidence of caspase-9 cleavage was confirmed by western analysis. Moreover, the timing of mitochondrial damage precedes the cleavage of caspase-9 as presented in this report. Although the activation of an apoptotic response by the initiator caspase-8, -9 and executioner caspase-3 in response to BTV replication has been reported previously, to date the relationship between the two pathways remains unknown.

In this study, the relationship between the intrinsic and extrinsic pathways was determined using a series of assays including cell markers, pharmacological inhibitors and knock-out cell lines. Unlike reoviruses [35], there was no cleavage of BID by caspase-8 in BTV infected cells. Therefore, the extrinsic pathway is unlikely to induce the mitochondrial damage as observed in our studies. Furthermore, the use of a chemical inhibitor and the caspase-9 deficient cell line (Jurkat ΔC9) clearly confirmed that caspase-8 activation was also independent of caspase-9 during BTV infection of mammalian cells. Thus, the activation of each caspase pathway during BTV-1 infection appears to be independent.

In addition to the activation of executioner caspase-3, we found that BTV infection also induces the activation of caspase-7, another executioner caspase which is closely related to caspase-3, sharing similar structure and substrates, but less promiscuous [36]. Caspase-3 and -7 share a number of target proteins and both recognise caspase death substrate PARP. We investigated PARP cleavage in BTV infection and the cleavage of PARP was detected in response to BTV infection of mammalian cells, adding further support that BT clinical signs are mainly mediated by apoptosis.

DeMaula *et al. *[5,6] have hypothesised that the main component of BT clinical signs and lesions in endothelial cells is due to cellular necrosis. Their hypothesis was based on the observation at the late stage of BTV infection. In this study, we examined cellular necrosis by using a cellular marker HMGB-1. When necrosis is induced it causes the loss of cellular membrane integrity and HMGB-1 is translocated from nucleus to cytoplasm and rapidly released into the extracellular space, which results in inflammation and tissue damage [22,37,38]. When we examined the cytosolic and nuclear fractions of BTV infected HeLa at 48 h p.i., there was no HMGB-1 translocation to the cytosol. Therefore, the cell death and damage observed in BTV infection of mammalian cells is not due to necrosis but probably due to the very late timing of events and the cell disruption caused by the inflammatory response.

The data presented in our paper demonstrates that BTV infection of mammalian cells induces caspase cascade, resulting in apoptosis. These results indicate that apoptosis is a major cause of cellular damage in the host animal, supporting previous reports [9,12]. However, it will be imperative to investigate the caspase activation and the role of apoptosis in BT disease in susceptible sheep.

Previously, we reported the activation of NF-κB in BTV infected cells [11] and hypothesised that it has a role in BTV-induced apoptosis similar to that of reovirus T3 [13]. However, here we found that the inhibition of NF-κB had no effect in virus-induced apoptosis. Indeed, our data clearly demonstrated that chemical inhibition of the p50 subunit of NF-κB enhanced the early onset of visible cytopathic effect in BTV infected cells, and thus contradicted the report of Mortola and Larsson [25].

Therefore, it was necessary to further investigate the response of NF-κB to BTV infection. The type of response by NF-κB is related to the phosphorylation and degradation of the IκB complex, an inhibitory protein complex, which masks the nuclear translocation signal of NF-κB. Our data showed the degradation of IκBα but not IκBβ in response to BTV infection, which indicates that the classical NF-κB pathway is activated resulting in a controlled transient response. Furthermore, we examine the activation of NF-κB by using a NF-κB dependent firefly luciferase reporter assay. Our data showed an initial early period of NF-κB activation that was not sustained, as the NF-κB activity appeared to be inhibited at later stages of virus replication. The NF-κB response observed by BTV infection was similar to that of the T3 strain of reovirus [39].

From our preliminary data, we postulate that the transient nature of the NF-κB response was due to BTV proteins suppression of NF-κB activation, which in turn allowed efficient virus replication. BTV NS1 and NS2 limited NF-κB activation when the response was stimulated by poly I:C. Both of these proteins have the ability to bind RNA and therefore could act by sequestering dsRNA and inhibiting the pathway, or could interact with cellular proteins to inhibit the cascade (i.e. rotavirus [40]), or inhibit mRNA export from the nucleus (i.e. rotavirus NSP3 [41]).

Thus, our data showed that activation of NF-κB by BTV infection had a minimal role, if any, in the induction of apoptosis. However, it may play a role in initiating an antiviral state through the induction of the innate immune response. To this end, we investigated the effect of NF-κB activation on BTV replication and generation of infectious virions. In the presence of an NF-κB inhibitor, BTV titres were higher at the early stages (up to 24 h p.i.) of virus replication than in the absence of inhibitor. This result would indicate that NF-κB activation acts to control virus replication. At the late times, virus titres were similar in both cases, which could be indicative of the virus proteins controlling the NF-κB response (i.e. NS1 and NS2). The induction of NF-κB response in controlling virus replication through the induction of an antiviral response needs to be investigated further, especially as a recent report demonstrated that BTV replication was significantly high in IFNα deficient mice resulting in rapid death of these animals [42].

BTV infection has been reported to produce strong cytokine responses [7,43-45], however the mechanisms that trigger their production has not been investigated. NF-κB activation could be a candidate to initiate a cytokine response during BTV infection, although there are other pathways, including IRF that could induce cytokine response. In this report we were able to identify the translocation of IRF-3 to the nucleus in response to BTV infection, indicating the induction of IRF-3 by BTV infection. As well as IRF-3, which is sequestered in the cytosol, the production of IRF-7 and its subsequent translocation to the nucleus were also detectable in BTV infected cells. Moreover, using an IRF dependent firefly luciferase reporter assay we confirmed that IRF activation was stimulated during BTV infection. Interestingly, the kinetics of IRF activation mirrored NF-κB activation. This early response of IRF was suppressed over the course of BTV infection similar to that of NF-κB. Despite the activation of IRF and NF-κB, it is clear that BTV infection triggers robust apoptosis in mammalian cells, which most likely play a role in BTV pathogenesis.

## Materials and methods

### Cells, viruses and antibodies

Human cervical epithelial carcinoma (HeLa) and BSR cells were maintained in Dulbecco's modified Eagle's medium (DMEM; Lonza) supplemented with 10% fetal calf serum (FCS), 100 U/ml penicillin and 100 mg/ml of streptomycin (Lonza) and incubated at 35°C, with CO_2_. Jurkat T-cells were purchased from ATCC and caspase-9 deficient Jurkat T-cells (Jurkat ΔC9) and complemented caspase-9 Jurkat T-cells (Jurkat compΔC9) were kindly provided by Klaus Schulze-Osthoff (University of Dusseldorf, Germany). The cells were grown in RPMI media (Sigma Aldrich) supplemented with 6% FCS, 100 U/ml penicillin and 100 mg/ml of streptomycin (Lonza) and incubated at 35°C, with CO_2_.

BTV serotype 1 (BTV-1) was titred by plaque assay as described [46] and multiplicity of infections (MOI) between 0.1 and 1. For all experimental infections, cell monolayers were washed with FCS free growth medium, incubated with BTV-1 at the appropriate MOI in serum-free medium for 30 mins, the virus inoculums were aspirated, the cells washed twice in excess DMEM and further incubated in DMEM supplemented with 2% FCS and 100 U/ml penicillin and 100 mg/ml of streptomycin for the time course of the experiment. The procedure was similar for the infection of Jurkat T-cells with the exception that RPMI media was used.

Antibodies for detection of the caspase-8 (ab25901), caspase-9 (ab25758), caspase-3 (ab17819; ab90437) and anti-HMGB were purchased from Abcam. Active caspase-8 (18C8 #9496), caspase-9 (#9501) and caspase-7 (#9492) antibodies were purchase from Cell Signaling. Antibodies to detect the cellular proteins (tubulin and actin; AC-15), caspase-3 (clone 4H334) and PARP (clone C-2-10) were purchased from Sigma Aldrich. Specific antibodies for BTV were generated in house. Inhibitors of caspase cleavage were purchased from Calbiochem and Sigma Aldrich. Anti-IkBα (E:130), anti-IkBβ (ab7547), anti-NF-κB (p50/p105) and anti-NF-κB (p65) polyclonal antibodies were purchased from Abcam. Inhibitor of NF-κB (SN50; Calbiochem) was resuspended in distilled water was used at final concentration of 32 uM. Anti-IRF-3 (FL-425, sc9082) and anti-IRF-7 (H-246, sc9083) polyclonal antibodies were purchased from Santa Cruz.

Alkaline phophatase conjugated secondary antibodies were purchased from Millipore (mouse) and Sigma Aldrich (guinea pig and rabbit). Tetramethyl rhodamine isothiocyanate (TRITC) and fluorescein isothiocyanate (FITC)-conjugated secondary antibodies were purchased from Sigma Aldrich.

### Preparation of cell lysates for the immunoblot

Whole cells (detached and adhered) were harvested and rinsed with cold 150 mM phosphate-buffered saline (PBS) and resuspended in SDS PAGE lysis buffer and processed for western immunoblot. For preparation of different cellular fractions, cells were harvested and incubated in Hepes buffer, pH 6.8 (10 mM Hepes 10 mM KCl, 1.5 mM Mg_2_Cl, 340 mM sucrose, 10%(v/v) glycerol, 0.1%(w/v) Triton X-100, 1× protease-inhibitor cocktail and 10 μM phenylmethylsulfonyl fluoride (PMSF) on ice for 30 min and then centrifuged at 4°C, 5 min at 3 000 xg (nuclear fraction). The supernatant was further centrifuged at 4°C for 45 min at 20 000 xg to separate the cytosolic fraction from the pellet (mitochondria) and the cell extracts were analysed by western immunoblot.

### Western immunoblot analysis

The proteins were resolved on 10-15% SDS-PAGE gels according to target protein molecular weight, and transferred to a nitrocellulose membrane (0.45 micron, Amersham) by the standard semi-dry transfer protocol. Each blot was developed with specific primary and secondary antibodies following the manufacturer protocols.

### Immunofluorscence by confocal microscopy

BTV-infected cells were fixed with 4% (w/v) paraformaldehyde on coverslips, washed in PBS, permeabilised and blocked as described [47]. Subsequently, cells were immuno-labelled with primary antibodies, diluted (1:100-1:200), washed and incubated with appropriate secondary antibodies conjugated to TRITC (1:64) or FITC (1:128). Cover slip were mounted in Vectashield mounting media (Vector Laboratories, Burlingham) and were examined with a Zeiss Axiovert 200 M laser-scanning microscope fitted with a helium-argon laser. Images were acquired and analysed using LSM 510 confocal software (Zeiss).

### NFκB and IRF dependent luciferase reporter gene assays

The NF-κB dependent luciferase reporter plasmid, pGL3NF-κB, was constructed by ligating the 4 repeats of the of the decameric sequence (GGGAATTTCC) recognised by NF-κB into the *Nde*1-*Bgl*II sites of pGL3 basic luciferase. The IRF dependent luciferase reporter plasmids were kindly provided by Takashi Futija (Kyoto University, Japan) and were previously described [48,49].

HeLa cells grown in 96 well plates were transfected with 0.1 μg/well of the reporter using Lipofectamine2000™ according to manufacturer's protocols. At 24 h post transfection, cells were either infected with BTV-1 or transfected with 1 μg/well of poly I:C (Amersham Pharmacia) using Oligofectamine™ as per manufacturer's protocol or treated with 0.6 μg/ml DOX (doxorubicin.HCl; Sigma Aldrich) or mock infected. Each treatment was undertaken in triplicates and the assay performed no less than 5 times. The firefly luciferase activity in each well was quantified at different times using the Dual-Luciferase Assay Kit (Promega) to detect both firefly and Renilla luciferase.

For the inhibition assays using NF-κB (SN50) inhibitor, cells were transfected with the reporter plasmid 24 h prior to treatment with 32 μM SN50 as above and then either infected with BTV, or transfected with poly I:C or treated with DOX.

To study the effect of the BTV proteins on the NF-κB or IRF activation, HeLa cells grown in 96-well plates were co-transfected with 25 ng of T7 generated BTV transcripts or Renilla transcript with either the NF-κB or IRF dependent reporter construct using Lipofectamine2000™. T7 mRNA transcripts for VP2, VP4, VP6 and NS1-3 were generated from the BTV-1 exact copy constructs as previously described [50]. At 24 h post transfection, the NF-κB or IRF response was induced by transfecting the cells with 1 μg poly I:C. The firefly luciferase activity was measured 6 and 24 h post induction with the Dual-Luciferase assay kit (Promega).

### Virus titration by TCID_50_/ml

The growth of BTV in the presence of NF-κB (SN50) inhibitor was determined using TCID_50 _(tissue culture infectious dose of 50%) endpoint titration. BSR were grown in 96-well plates and 10-fold serial dilution of the BTV at different times p.i. were made in DMEM supplemented with 2% FCS in replicates of 5. The cells were fixed with 1% (w/v) paraformaldehyde, CPE was visualised by crystal violet stain and the titre determined as a TCID_50_/ml.

## Competing interests

The authors declare that they have no competing interests.

## Authors' contributions

PR conceived the project and designed experiments. MS designed experiments and carried out research. PR and MS wrote the manuscript and prepared the figures. Both authors approved the final manuscript.
